# Reliability and validity of the MX3 portable sweat sodium analyser during exercise in warm conditions

**DOI:** 10.1007/s00421-024-05447-w

**Published:** 2024-03-02

**Authors:** Harry A. Brown, Brad Clark, Julien D. Périard

**Affiliations:** Research Institute for Sport and Exercise Science (UCRISE), University of Canberra, Bruce, Canberra, ACT Australia

**Keywords:** Heat, Hydration, Electrolytes, Performance

## Abstract

**Purpose:**

Accurately measuring sweat sodium concentration ([Na^+^]) in the field is advantageous for coaches, scientists, and dieticians looking to tailor hydration strategies. The MX3 hydration testing system is a new portable analyser that uses pre-calibrated biosensors to measure sweat [Na^+^]. This study aimed to assess the validity and reliability of the MX3 hydration testing system.

**Methods:**

Thirty-one (11 females) recreationally active participants completed one experimental trial. During this trial, participants exercised at a self-selected pace for 45 min in a warm environment (31.5 ± 0.8 °C, 63.2 ± 1.3% relative humidity). Sweat samples were collected from three measurement sites using absorbent patches. The samples were then analysed for sweat [Na^+^] using both the MX3 hydration testing system and the Horiba LAQUAtwin-NA-11. The reliability of the MX3 hydration testing system was determined following two measurements of the same sweat sample.

**Results:**

The mean difference between measurements was 0.1 mmoL·L^−1^ (95% limits of agreement (LoA): − 9.2, 9.4). The analyser demonstrated a coefficient of variation (CV) of 5.6% and the standard error of measurement was 3.3 mmoL·L^−1^. When compared to the Horiba LAQUAtwin-NA-11, there was a mean difference of − 1.7 mmoL·L^−1^ (95% LoA: − 0.25${\overline{\text{X}}}$, 0.25${\overline{\text{X}}}$) and the CV was 9.8%.

**Conclusion:**

The MX3 hydration testing system demonstrated very good single-trial reliability, moderate agreement and a very good CV relative to the Horiba LAQUAtwin-Na-11. To further validate its performance, the MX3 hydration testing system should be compared with analytical techniques known for superior reliability and validity.

## Introduction

During exercise in hot and/or humid environmental conditions, whole-body sweating leads to the loss of several electrolytes including sodium, chloride, and potassium (Sawka and Montain 2000; Périard et al. 2021). Sodium in particular, is purported to be key for maintaining plasma osmolality and regulating fluid movement between the intra- and extra-cellular compartments (Baker and Wolfe 2020). The concentration of sweat sodium ([Na^+^]) has been documented to vary significantly between individuals, ranging from as low as 10 mmoL·L^−1^ to exceeding 100 mmoL·L^−1^ (Baker et al. 2022; Sawka et al. 2007). The variability in sweat composition is associated with several factors such as exercise intensity (Baker et al. 2022), acclimation status (Kirby and Convertino 1986), sex (Lobeck and Huebner 1962) and dietary sodium intake (Allsopp et al. 1998). Consequently, it is recommended that athletes adopt personalised hydration strategies, often incorporating sodium replenishment, so as to reduce the risk of exercise-induced hypohydration, heat cramps, and decrements in exercise performance (Sawka et al. 2007).

When measuring sweat [Na^+^], the whole-body washdown technique is proposed to provide the greatest accuracy (Baker 2017). However, this technique is most appropriate in a laboratory environment when individuals are sweating passively or during stationary cycling (Shirreffs and Maughan 1997). Accordingly, there has been an increase in demand for local sweat [Na^+^] measurement techniques that can be conducted in the field as surrogates for the whole-body washdown technique. Current field techniques include absorbent patches and compact analysers capable of accurately and reliably measuring local sweat [Na^+^] (Goulet et al. 2012; Dziedzic et al. 2014). Such analytical techniques allow coaches, athletes, and occupational workers to measure sweat [Na^+^] and tailor hydration strategies to suit the specific needs of individuals whilst remaining in the field.

A new compact analyser on the market is the MX3 hydration testing system. The MX3 system has previously been used to assess hydration status via salivary osmolality (Faidah et al. 2021). More recently, MX3 have adapted the analyser so that the device can also measure sweat [Na^+^] using pre-calibrated biosensors for ease of use. A unique element of the MX3 system is the link to an associated software program where each analyser measurement is documented and allows users to create an athlete profile and track repeated measurements. This tracking is proposed to aid coaches, scientists, and dieticians to individualise hydration strategies. However, an independent validation of the MX3 sweat [Na^+^] analyser has yet to be undertaken. Therefore, the aim of this study was to determine the validity and reliability of the MX3 hydration testing system when measuring sweat [Na^+^]. The accuracy of the system was compared to the Horiba LAQUAtwin-Na-11, another portable sweat [Na^+^] analyser designed to be used in the field that has been shown to be reliable with acceptable relative and absolute validity (Goulet et al. 2017).

## Methods

### Participants

Thirty-one (11 females) recreationally active adults volunteered for this study (McKay et al. 2022). Participants’ mean age, height, and weight were 28 ± 7 yr, 175 ± 8 cm and 73.5 ± 12.0 kg, respectively. All participants completed a single trial between June and September (i.e. southern hemisphere winter/early spring in Canberra, Australia) and as such, were assumed to be non-heat acclimatised (Brown et al. 2022). All participants provided informed consent prior to participating in this study, which was approved by the University of Canberra Research Ethics Committee (Project ID: 12199) and conformed to the *Declaration of Helsinki.*

## Experimental procedure

On arrival at the laboratory, participants exercise clothing was weighed and they then provided a urine sample to assess urine-specific gravity (USG: PEN-Urine S.G.; Atago Co. Ltd., Tokyo, Japan). If USG was > 1.025, participants were provided with 5 mL∙kg^−1^ of water to consume ~ 30 min before the commencement of exercise. Height and body mass (wearing the clothes that had been weighed) were then measured with a wall-mounted stadiometer (Seca 220, Germany) and digital scale (KW Industrial platform scales, Atweigh, Victoria, Australia), respectively. Thereafter, participants were fitted with a heart rate monitor chest strap (HRM Dual, Garmin Ltd, Olathe, Kansas, USA) and three absorbent patches (5 × 7 cm; Tegaderm, 3 M, USA). The three absorbent patches were fitted to the left ventral forearm, upper back (superior to the right scapula, ~ 15 cm lateral of the vertebral column) and middle of the right thigh (anterior).

Participants then entered an environmental chamber set to warm conditions (31.5 ± 0.8 °C, 63.2 ± 1.3% relative humidity) without artificial convection (i.e. no fanning) and mounted a motorised treadmill (Pulsar 3p, H/P/Cosmos, Nussdorf-Traunstein, Germany). They then completed 45 min of running at a self-selected speed equivalent to 11 (“Light”) on the 6–20 rating of perceived exertion (RPE) scale (Borg 1982) for the first 5 min, then at a speed equivalent to 13 (“Somewhat hard”) on the RPE scale for the remaining 40 min. Treadmill speed was adjusted throughout the run by the participants to maintain the prescribed RPE. Environmental conditions and heart rate were monitored throughout the trial. On the completion of exercise, participants were weighed, after which their clothes were weighed separately, and whole-body sweat loss was calculated (Cheuvront and Kenefick 2017).

### Sweat sample analysis and preparation

Before fitting the patches, the three sites were cleaned with deionised water and wiped dry to remove any potential sample contaminants. When necessary, sites were shaved to remove hair and then rinsed and dried before the placement of the patches. Following preparation of the skin sites, each patch was put in place and pressed firmly on the skin for ~ 5 s. On completion of the trial, participants left the environmental chamber and sat resting on a chair before patches were removed using sterilised tweezers. The patches were immediately placed into a 5 mL syringe and analysed using both portable sodium analysers (MX3 Diagnostics Inc., Melbourne, Australia; LAQUAtwin-Na-11, Horiba Advanced Techno, Co., Ltd., Kyoto, Japan). All sweat samples were analysed in a temperate environment (~ 22 °C).

### MX3 hydration testing system

The MX3 hydration testing system is a portable (~ 160 g, 214 mm × 45 mm × 25 mm), battery operated (Type Single Cell rechargeable, Li-Po, 3.7 V, 1100mAh) analyser, capable of being used for hydration status testing based on salivary osmolarity and for sweat [Na^+^] analysis. The portable system uses pre-calibrated biosensor strips to determine sweat [Na^+^]. Each sample was measured by tapping the pre-calibrated biosensor strip directly on to the sweat sample that had been transferred into a tray (4.5 cm × 4.5 cm). This procedure was completed in triplicate before the portable system provided an average (in mg∙L^−1^) of the three strips. Each single measurement (i.e. one strip) took ~ 10 s and the average value was calculated within the application immediately following a successful measurement from the third strip. When one of the three biosensor strips recorded a value outside the acceptable range of the other two, leading to a CV of > 10%, the analyser prompted a fourth, and potentially fifth sample to be recorded. To determine system reliability, a further three pre-calibrated biosensor strips were used to generate a second average. This process was completed for each of the samples from the three absorbent patch sites.

### Horiba LAQUAtwin-Na-11

The LAQUAtwin-Na-11 was calibrated using instructions provided by the manufacturer, as published previously (Goulet et al. 2017). Once turned on, the sensor was cleaned using deionised water and then wiped using a sterile gauze. Thereafter, a two-point calibration was performed using 150 and 2000 ppm standards. The sensor was then cleaned again using deionised water and a sterile gauze. This cleaning procedure was repeated following each sample placed onto the sensor directly from the syringe, and the entire calibration process was repeated before each trial. Sample measurements were recorded when the displayed value met the stability criteria of the analyser and a “smiley face” icon appeared. The Horiba LAQUAtwin-Na-11 is the updated version of a previously validated sweat [Na^+^] analyser (Horiba B-722 LAQUAtwin; Goulet et al. (2017)).

### Statistical analyses

All analyses were conducted in R statistical software (v 4.2.1) (R Core Team 2022). As the typical reporting of sweat [Na^+^] is in mmoL·L^−1^, the measurements from both analysers were converted from mg∙L^−1^ to mmoL·L^−1^ before any analysis. The reliability of the MX3 hydration testing system was evaluated using the duplicate values from each of the three sites and determined using the mean difference, coefficient of variation (CV), standard error of the measurement, intraclass correlation coefficient, and 95% limit of agreements (LoA).

The validity of the MX3 Hydration testing system was evaluated using measurements from the LAQUAtwin-Na-11 of sweat collected from the same absorbent patch. Validity of the MX3 hydration testing system was determined by calculating the mean difference, CV, 95% LoA, and concordance correlation with the LAQUAtwin-Na-11 measurement. Analyses were conducted using the ‘SimplyAgree’ package (Caldwell 2022). On visual inspection of the data comparing the two analysers, the error appeared to increase as the measured value increased, which suggested that the data were heteroscedastic. As such, before the validity analysis, data was log-transformed, the LoA were calculated, and then transformed back to their original scale (i.e. mmoL·L^−1^) for ease of interpretation by taking the anti-logs (Euser et al. 2008). To further aid the interpretation of the LoA, data are provided in a ratio scale and as a function of the mean X̄ (Bland and Altman 1996; Euser et al. 2008). The CV was interpreted as: very good (< 10%), good (10–20%), acceptable (20–30%), and not acceptable (> 30%) (Walters et al. 2021). The concordance correlation coefficient was interpreted as: poor (< 0.90), moderate (0.90–0.95), substantial (0.95–0.99) and almost perfect (> 0.99) (McBride 2005). Data were visualised as correlation and Bland–Altman plots using the ‘ggplot2’ package (Wickham 2016).

## Results

Participant pre-exercise USG was 1.014 ± 0.008. During exercise, mean heart rate was 165 ± 10 beats∙min^−1^ (range: 146–184 beats∙min^−1^). Participants ran an average of 6.9 ± 1.3 km (range: 4.1–9.3 km) and whole-body sweat loss was 0.87 ± 0.34 L (range: 0.32–1.85 L).

### Reliability

**Fig. 1:**
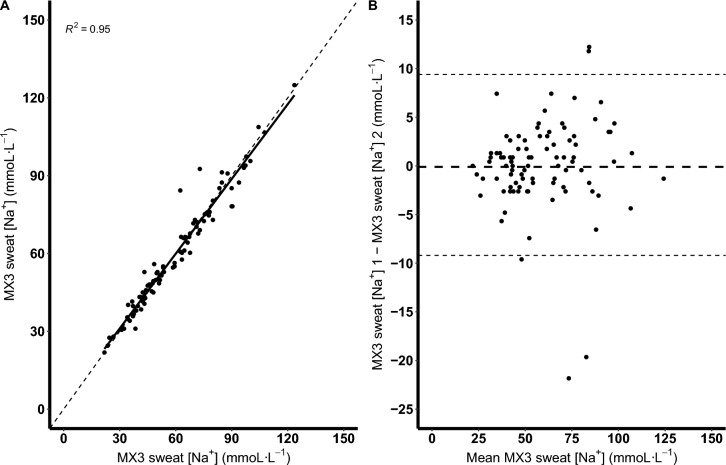
Correlation (sold line) plot between the first and second measurement from the MX3 hydration test system across all sites (**A**); dashed line represents the line of identity. Bland–Altman plot showing the mean difference (thick black dashed line) and the 95% limits of agreement (thin black dashed lines) of the first and second readings from the MX3 hydration test system (**B**)

The reliability of the MX3 hydration testing system is shown in Table 1 and Fig 1A. Across the three measurement sites, the mean difference between the first and second measurements was 0.1 mmoL·L^−1^ (95% LoA: − 9.2, 9.4; Fig. 1B). The CV was 5.6% and the standard error of the measurement was 3.3 mmoL·L^−1^. The highest sweat [Na^+^] measurements were from the back, which was also the site with the largest CV (6.6%) and lowest intraclass coefficient (0.958). Duplicate measurements using the Horiba LAQUAtwin-Na-11 were completed for 55 sweat samples, but insufficient sweat prevented duplicate measurements for the remaining 26 samples. The Horiba LAQUAtwin-Na-11 demonstrated a mean difference between measurements of 0.5 mmoL·L^−1^ (95% LoA: − 6.6, 7.6), a CV of 4.2% and a standard error of the measurement of 2.5 mmoL·L^−1^.

**Table 1:** Reliability of the MX3 hydration testing system for measuring sweat [Na^+^]

	All sites		Arm		Back		Thigh	
Variable	Measurement 1	Measurement 2	Measurement 1	Measurement 2	Measurement 1	Measurement 2	Measurement 1	Measurement 2
No. of samples	91	91	30	30	31	31	30	30
Mean ± SD (mmoL·L^−1^)	59.2 ± 21.8	59.2 ± 21.4	56.7 ± 23.4	57.4 ± 23.1	68.3 ± 22.1	68.0 ± 22.0	52.4 ± 16.5	51.8 ± 15.6
Range (mmoL·L^−1^)	21.8–123.6	21.8–124.9	21.8–123.6	21.8–124.9	24.5–107.9	27.5–108.7	23.6–90.4	24.5–87.3
Mean difference (mmoL·L^−1^)	0.1		− 0.7		0.3		0.7	
95% LoA	− 9.2, 9.4		− 8.3, 6.8		− 12.5, 13.0		− 5.6, 7.0	
SEM (mmoL·L^−1^)	3.3		2.7		4.5		2.3	
CV (%)	5.6		4.8		6.6		4.4	
ICC (r)	0.976		0.986		0.958		0.980	

*CV* coefficient of variation, *ICC* interclass correlation coefficient, *LoA* Limits of Agreement, *SD* standard deviation, *SEM* standard error of measurement

### Validity

**Fig. 2:**
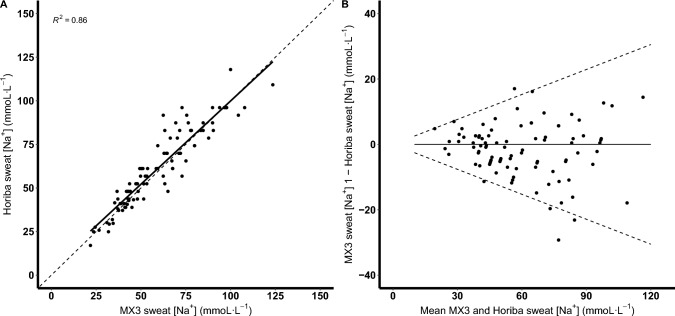
Correlation (solid line) plots between the MX3 hydration testing system and the Horiba LAQUAtwin-Na-11 across the three measurement sites for sweat [Na.^+^] (**A**); dashed line represents the line of identity. Bland–Altman plots showing the mean difference (thick black dashed line) and the 95% limits of agreement (thin black dashed lines) between the MX3 hydration testing system and the Horiba LAQUAtwin-Na-11 (**B**)

A comparison of sweat [Na^+^] between the MX3 hydration testing system and Horiba LAQUAtwin-Na-11 is shown in Table 2 and Fig. 2A and B. Across all sites, the mean difference between the MX3 hydration testing system and Horiba LAQUAtwin-Na-11 was − 1.7 mmoL·L^−1^ (95% LoA: − 0.25${\overline{\text{X}}}$, 0.25${\overline{\text{X}}}$), and a CV of 9.8%. Sweat [Na^+^] measurements from the back demonstrated the least agreement with a mean difference of − 5.1 mmoL·L^−1^. For sweat [Na^+^] measurements, the MX3 hydration testing system determined to be under 60 mmoL·L^−1^ (*n* = 50), the mean difference to the Horiba LAQUAtwin-Na-11 was − 2.1 (95% LoA: − 12.1, 8.0) with a CV of 8.3%. However, for sweat [Na^+^] measurements, the MX3 hydration testing system determined to be greater than 60 mmoL·L^−1^ (*n* = 41), the mean difference to the Horiba LAQUAtwin-Na-11 was − 1.3 mmoL·L^−1^ (95% LoA: − 23.0, 20.4) with a CV of 9.7%. For sweat [Na^+^] measurements, the MX3 hydration testing system determined to be under 60 mmoL·L^−1^, there was a 0.862 concordance correlation coefficient between the two analysers. For sweat [Na^+^] measurements, the MX3 hydration testing system determined to be greater than 60 mmoL·L^−1^, there was a 0.724 concordance correlation coefficient with the Horiba LAQUAtwin-Na-11.

**Table 2:** Comparison of the MX3 hydration test system with the Horiba LAQUAtwin-Na-11 for measuring sweat [Na^+^]

	MX3-Horiba			
Variable	All sites	Arm	Back	Thigh
No. of samples	91	30	31	30
Mean difference (mmoL·L^−1^)	− 1.7	3.7	− 5.1	− 3.7
Ratio 95% LoA	0.76, 1.26	0.87, 1.33	0.73, 1.18	0.77, 1.13
95% LoA as function of the mean X̄	− 0.25${\overline{\text{X}}}$, 0.25${\overline{\text{X}}}$	− 0.21${\overline{\text{X}}}$, 0.21${\overline{\text{X}}}$	− 0.24${\overline{\text{X}}}$, 0.24${\overline{\text{X}}}$	− 0.19${\overline{\text{X}}}$, 0.19${\overline{\text{X}}}$
CV (%)	9.8	8.5	9.5	7.2
CCC (r)	0.926	0.946	0.884	0.921

*CCC* concordance correlation coefficient, *CV* coefficient of variation, *LoA* Limits of Agreement

## Discussion

The aim of this study was to test the reliability and validity of the MX3 hydration testing system, a new, portable sweat [Na^+^] analyser. The reliability of the MX3 hydration testing system was determined by measuring sweat samples collected from three measurements sites in duplicate following a 45 min treadmill run in a warm environment. The validity of the analyser was then established by comparing it to that of the Horiba LAQUAtwin-Na-11 (Goulet et al. 2017). Our results show that the MX3 hydration testing system has high single-trial reliability for measuring sweat [Na^+^], with a mean difference of 0.1 mmoL·L^−1^ (95% LoA: − 9.2, 9.4) between duplicate measures of 91 independent sweat samples. In addition, the MX3 hydration testing system had a 5.6% CV, which is deemed to be ‘very good’ (Walters et al. 2021), and the standard error of the measurement was 3.3 mmoL·L^−1^. The MX3 hydration testing system demonstrated a smaller mean difference than the Horiba LAQUAtwin-Na-11 in their respective validity study of a previous version; however, the Horiba LAQUAtwin-Na-11 demonstrated a smaller CV (2.6%) (Goulet et al. 2017). Importantly, given the small difference and very good CV between the first and second measurement of sweat [Na^+^], the MX3 hydration testing system can be deemed reliable to detect changes in sweat [Na^+^] during repeated measures in the same participant. Moreover, 95% LoA of the MX3 hydration testing system suggests that it is sufficiently accurate to detect adaptations associated with both heat acclimatisation (i.e. ~ 15 mmoL·L^−1^; Brown et al. (2023)) and heat acclimation (i.e. ~ 22 mmoL·L^−1^; Tyler et al. (2016)), as well as changes in sweat [Na^+^] associated with the development of hyponatremia (i.e. ~ 25 mmoL·L^−1^; Montain et al. (2006)). This is an important consideration for coaches and researchers wanting to use a portable analyser to evaluate within participant changes or adaptations (e.g. pre- *vs*. post-heat acclimation).

Compared to the Horiba LAQUAtwin-Na-11, the MX3 hydration testing system provided measures yielding a mean difference in sweat [Na^+^] of − 1.7 mmoL·L^−1^ (95% LoA: − 0.25X̄, 0.25X̄), in addition to a CV of 9.8%. In the previously published validation trial of a Horiba LAQUAtwin analyser, there was a mean difference of 1.3 mmoL·L^−1^, a CV of 3.9% and standard error of the estimate of 3.8 mmoL·L^−1^ compared to a high performance ion chromatograph (Goulet et al. 2017). As shown in Fig. 2Aand 2B, the reported difference between measurements from the two analysers appeared to be greater after ~ 60 mmoL·L^−1^, which is supported by the differences in concordance correlation coefficient between the analysers above and below 60 mmoL·L^−1^ (i.e. 0.862 *vs* 0.724). This may indicate an inherent error (i.e. heteroscedasticity) in the MX3 hydration testing system at higher sweat [Na^+^]. However, it is important to note that for measurements > 1000 ppm (i.e. ~ 44 mmoL·L^−1^), the Horiba LAQUAtwin-Na-11 rounds out measurements to the nearest 100 ppm (i.e. ~ 4 mmoL·L^−1^). This likely introduces measurement bias and is potentially a reason as to why the MX3 hydration testing system and Horiba LAQUAtwin-Na-11 appeared to agree less at a higher sweat [Na^+^] (Fig. 2A, B).

The natural within-subject variation of sweat [Na^+^] is ~ 15% (Baker 2017). To account for this variation, some researchers choose to categorise sweat [Na^+^] (Dziedzic et al. 2014) using established clinical diagnostic criteria (i.e. high, moderate, low) (Wescor Incorporated 2014). If the measurements of sweat [Na^+^] in the current study were categorised using this criterion, the MX3 hydration testing system and the Horiba LAQUAtwin-Na-11 would disagree on the categorisation of 22 (~ 24%) of the sweat samples. As such, despite what appears to be good reliability of both the MX3 hydration testing system and the Horiba LAQUAtwin-Na-11, due to the inherent variability in their measurements, practitioners should not use analysers interchangeably (Goulet et al. 2017).

In conclusion, the MX3 hydration testing system demonstrated very good single-trial reliability for measuring sweat [Na^+^], evidenced by a CV of 5.6% and a mean difference of 0.1 mmoL·L^−1^ (95% LoA: − 9.2, 9.4). However, the CV is higher than the CV reported for the Horiba LAQUAtwin-Na-11 (2.6%) (Goulet et al. 2017) and the CV of the Horiba LAQUAtwin-Na-11 calculated during the current study (4.2%). The concordance correlation coefficient, a measure of both precision and accuracy, indicates that the MX3 hydration testing system has moderate agreement according to predetermined criteria (McBride 2005) with the Horiba LAQUAtwin-Na-11 for measuring sweat [Na^+^]. In addition, the CV between the two analysers (9.8%) can be interpreted as ‘very good’ (Walters et al. 2021). However, given the 95% LoA (− 0.25${\overline{\text{X}}}$, 0.25${\overline{\text{X}}}$) between the MX3 hydration testing system and Horiba LAQUAtwin-Na-11, we recommend further research comparing the MX3 hydration testing system to an analytical technique with superior reliability and validity (i.e. ion chromatographer) to demonstrate the robustness of its validity (e.g. Goulet et al. (2017)). Notwithstanding, it appears that the MX3 hydration testing system provides a reliable and valid analytical technique for measuring sweat [Na^+^] in field and laboratory settings for those intending to investigate acute responses and chronic adaptations.

## Data Availability

Data available from the corresponding author on reasonable request.

## Abbreviations

CV: Coefficient of variation
LoA: Limit of agreements
[Na^+^]: Sodium concentration
RPE: Rating of perceived exertion
USG: Urine-specific gravity
